# Use of a Defibrillator Magnet to Extract Multiple Ingested Sharp Iron Objects from the Stomach at Laparotomy

**DOI:** 10.51894/001c.5071

**Published:** 2016-10-24

**Authors:** Mark W. Jones

**Affiliations:** 1 Department of General Surgery, McLaren Greater Lansing

**Keywords:** laparotomy, magnet, foreign bodies, ingested

## Abstract

This paper describes a technique for safely removing sharp ingested ferrous-based objects from the stomach at the time of laparotomy. It consists of a case report of a patient with psychiatric issues. He presented to our emergency department on several occasions after eating multiple foreign objects. Due to the large amount of ingested items, they could not be removed via the endoscope, therefore requiring laparotomy. A serious issue presenting to the surgeon and surgical team is puncture of surgical gloves and possible injury to the operating staff’s hands during extraction of sharp objects. This technique describes using a defibrillator magnet placed into a sterile specimen bag that is then inserted into a gastrotomy incision to remove any iron-based ingested sharps. As many ingested sharps such as needles, tacks, nails, screws and pins are ferrous-based, this technique is very useful and efficient.

## CASE PRESENTATION

**Figure 1:**
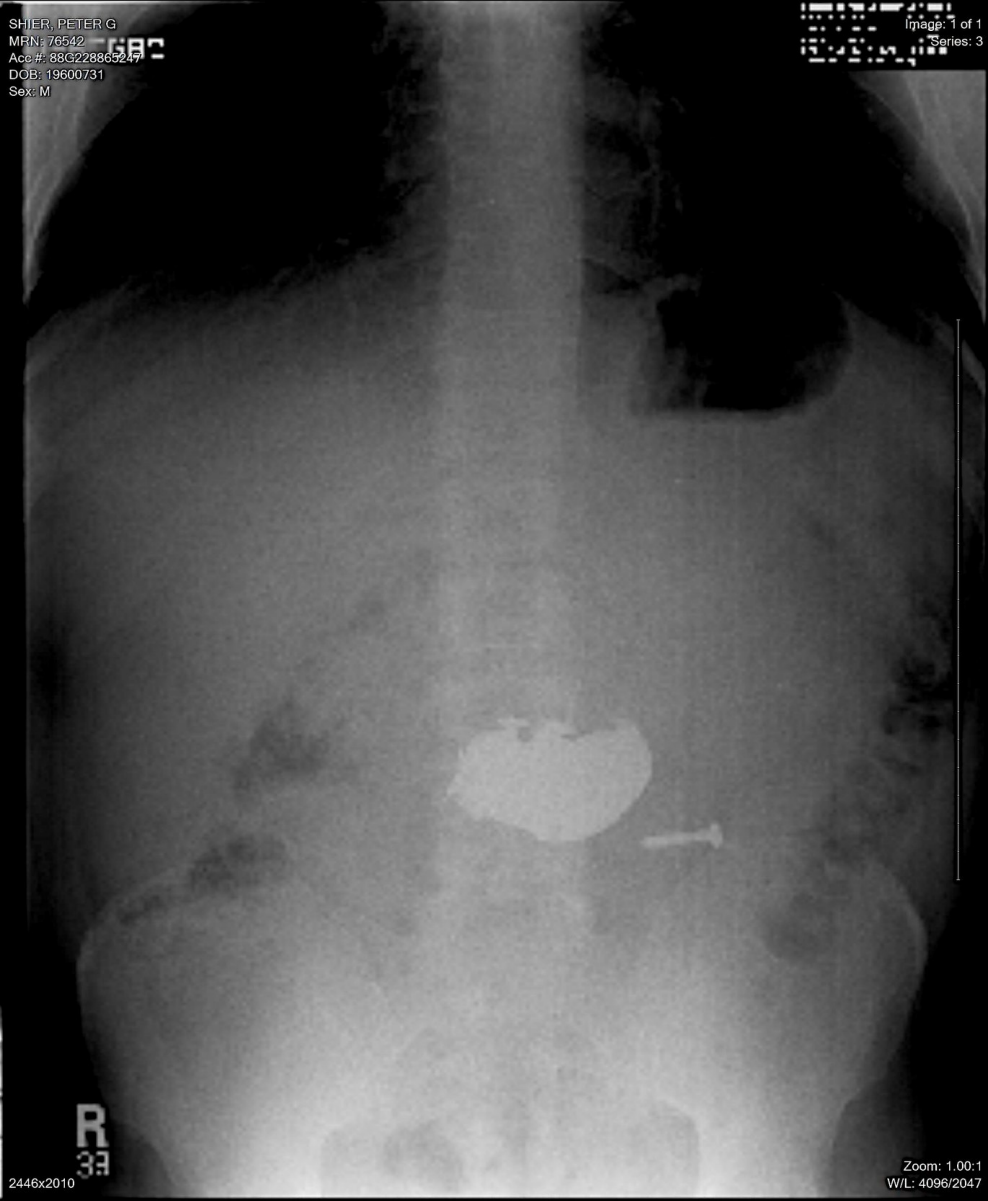
Upright abdominal x-ray demonstrating multiple ingested metallic objects.

A 51 yo male psychiatric patient suffering depression and alcohol abuse presented to our emergency department on two separate occasions after ingestion of large amounts of foreign materials. Figure 1 demonstrates an abdominal x-ray on one of his admissions. Presentation, physical exam and x-ray findings were almost identical on both admissions, as were the types of materials ingested. The large number of objects made endoscopic extraction impractical and, since the many sharps that were swallowed included nails and screws, surgical removal was required due to fear of perforation.

**Figure 2:**
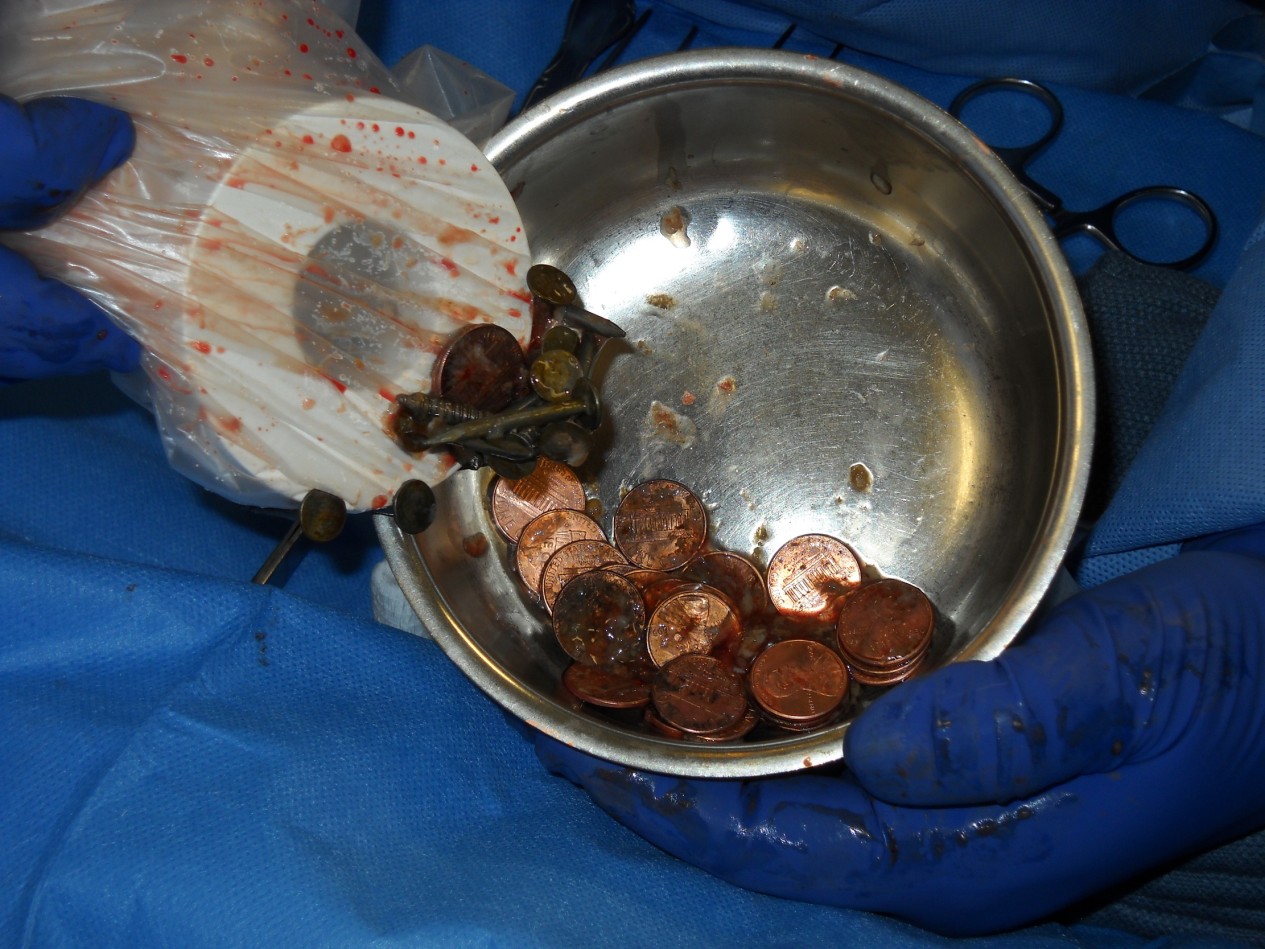
Retrieved sharps (nails and screws) clinging to defibrillator magnet at laparotomy

A defibrillator magnet (commonly found at the anesthesia station) was placed into a sterile specimen bag that was then inserted into a gastrotomy incision. The bag was tied at its opening to ensure sterility. As the magnet was inserted and pulled from the gastrotomy, the ferrous-based sharps clung to it, facilitating easy and safe removal (Figure 2). After all of the sharps were removed, the many remaining swallowed pennies were then safely scooped out with a gloved surgical hand. The small intestine was completely examined in surgery and no objects were found to have passed out of the stomach. This also was confirmed by a plain abdominal X-ray done post-operatively. The gastronomy was closed in a standard fashion. The patient had an uneventful post-operative course and was discharged 5 days after surgery.

This maneuver can be repeated as often as needed, and also works for objects that may be in the distal stomach or other harder to reach locations. This patient presented to our hospital two times with the same situation. He required a laparotomy on both occasions and this procedure worked effectively each time.

## DISCUSSION

Ingested foreign bodies can be sharp or pointed and pose a significant threat to patients, as well as to surgeons and staff who care for them. Puncture of surgical gloves and injury to the operating staff’s hands carries significant exposure issues. Many times these objects are iron-based, such as needles, tacks, nails, screws, pins and razor blades. If endoscopy is unsuccessful or not feasible, close observation may be attempted, often with good results. Conservative treatment usually consists of large amounts of daily fiber, possibly with oral mineral oil.^1^ The goal is for the patient to pass the objects without causing perforation or obstruction. Progress can be followed with serial x-rays.^2^ In this case the nature and large amount of ingested materials made conservative therapy prohibitive.

There are several papers that discuss the endoscopic use of magnets for removal of ingested iron objects. Fluoroscopic extraction utilizing specialized magnetized orogastric tubes also has been described.^3–5^ This is, however, the first paper we are aware of describing the use of a magnet for the extraction of ferrous-based objects at laparotomy. Many ingested items, especially sharp or pointed ones, are iron-based, making this technique useful in many situations. There is a myriad of other materials that can be eaten in which this technique will not be useful. Care must always be taken, as the true nature of swallowed objects is never known prior to surgery.

### Conflict of Interest

The author declares no conflict of interest.
